# Assessing Evidence for a Pervasive Alteration in Tropical Tree Communities

**DOI:** 10.1371/journal.pbio.0060045

**Published:** 2008-03-04

**Authors:** Jérôme Chave, Richard Condit, Helene C Muller-Landau, Sean C Thomas, Peter S Ashton, Sarayudh Bunyavejchewin, Leonardo L Co, Handanakere S Dattaraja, Stuart J Davies, Shameema Esufali, Corneille E. N Ewango, Kenneth J Feeley, Robin B Foster, Nimal Gunatilleke, Savitri Gunatilleke, Pamela Hall, Terese B Hart, Consuelo Hernández, Stephen P Hubbell, Akira Itoh, Somboon Kiratiprayoon, James V LaFrankie, Suzanne Loo de Lao, Jean-Rémy Makana, Md. Nur Supardi Noor, Abdul Rahman Kassim, Cristián Samper, Raman Sukumar, Hebbalalu S Suresh, Sylvester Tan, Jill Thompson, Ma. Dolores C Tongco, Renato Valencia, Martha Vallejo, Gorky Villa, Takuo Yamakura, Jess K Zimmerman, Elizabeth C Losos

**Affiliations:** 1 Laboratoire Evolution et Diversité Biologique CNRS/Université Paul Sabatier, Toulouse, France; 2 Center for Tropical Forest Science, Smithsonian Tropical Research Institute, Unit 0948, Panama; 3 Department of Ecology, Evolution, and Behavior, University of Minnesota, St. Paul, Minnesota, United States of America; 4 Faculty of Forestry, University of Toronto, Toronto, Ontario, Canada; 5 Center for Tropical Forest Science, Arnold Arboretum Asia Program, Harvard University Herbaria, Cambridge, Massachusetts, United States of America; 6 Thai National Park Wildlife and Plant Conservation Department, Chatuchak Bangkok, Thailand; 7 Institute of Biology, University of the Philippines, Quezon City, Philippines; 8 Center for Ecological Sciences, Indian Institute of Science, Bangalore, India; 9 Department of Botany, Faculty of Science, University of Peradeniya, Peradeniya, Sri Lanka; 10 Department of Botany, University of Missouri, St. Louis, Missouri, United States of America; 11 Department of Botany, Field Museum of Natural History, Chicago, Illinois, United States of America; 12 Department of Biology, Florida State University, Tallahassee, Florida, United States of America; 13 Wildlife Conservation Society, Bronx, New York, United States of America; 14 Laboratory of Plant Ecology, School of Biological Sciences, Catholic University of Ecuador, Quito, Ecuador; 15 Department of Plant Sciences, University of Georgia, Athens, Georgia, United States of America; 16 Plant Ecology Laboratory, Faculty of Science, Osaka City University, Osaka, Japan; 17 Faculty of Science and Technology, Thammasat University (Rangsit), Klongluang, Patumtani, Thailand; 18 Center for Tropical Forest Science, Asia Program, Nanyang Technological University, Singapore; 19 Forest Environment Division, Forest Research Institute Malaysia, Kepong, Kuala Lumpur, Malaysia; 20 National Museum of Natural History, Smithsonian Institution, Washington, D. C., United States of America; 21 Forest Research Center, Sarawak Forest Department, Kuching, Sarawak, Malaysia; 22 Institute for Tropical Ecosystem Studies University of Puerto Rico-Río Piedras, San Juan, Puerto Rico; 23 Instituto Alexander von Humboldt, Bogotá, Colombia; Imperial College, United Kingdom

## Abstract

In Amazonian tropical forests, recent studies have reported increases in aboveground biomass and in primary productivity, as well as shifts in plant species composition favouring fast-growing species over slow-growing ones. This pervasive alteration of mature tropical forests was attributed to global environmental change, such as an increase in atmospheric CO_2_ concentration, nutrient deposition, temperature, drought frequency, and/or irradiance. We used standardized, repeated measurements of over 2 million trees in ten large (16–52 ha each) forest plots on three continents to evaluate the generality of these findings across tropical forests. Aboveground biomass increased at seven of our ten plots, significantly so at four plots, and showed a large decrease at a single plot. Carbon accumulation pooled across sites was significant (+0.24 MgC ha^−1^ y^−1^, 95% confidence intervals [0.07, 0.39] MgC ha^−1^ y^−1^), but lower than reported previously for Amazonia. At three sites for which we had data for multiple census intervals, we found no concerted increase in biomass gain, in conflict with the increased productivity hypothesis. Over all ten plots, the fastest-growing quartile of species gained biomass (+0.33 [0.09, 0.55] % y^−1^) compared with the tree community as a whole (+0.15 % y^−1^); however, this significant trend was due to a single plot. Biomass of slow-growing species increased significantly when calculated over all plots (+0.21 [0.02, 0.37] % y^−1^), and in half of our plots when calculated individually. Our results do not support the hypothesis that fast-growing species are consistently increasing in dominance in tropical tree communities. Instead, they suggest that our plots may be simultaneously recovering from past disturbances and affected by changes in resource availability. More long-term studies are necessary to clarify the contribution of global change to the functioning of tropical forests.

## Author Summary

Recent studies have reported major changes in mature tropical forests, with increases in both forest biomass and net primary productivity, as well as shifts in plant species composition that favour fast-growing species over slow-growing ones. These pervasive alterations were attributed to global environmental change, and may result in dramatic shifts in the functioning of tropical forest ecosystems. We reassessed these findings using a dataset of large permanent forest plots on three continents. We found that tree biomass increased at seven of our ten plots, and showed a large decrease at a single plot. Overall, this increase was significant, albeit lower than reported previously for Amazonian forests. At three sites for which we had data for multiple census intervals, we found no concerted increase in biomass gain, in conflict with the increased productivity hypothesis. With the exception of one plot, slow-growing species gained more biomass than either fast-growing species or the tree community as a whole. Hence, our results do not support the hypothesis that fast-growing species are consistently increasing in dominance in tropical tree communities. Overall, our results suggest that our plots may be simultaneously recovering from past disturbances and affected by changes in resource availability.

## Abstract 3

An analysis of changes in biomass and primary productivity across ten large tropical forest plots revealed that aboveground biomass increased significantly, fast-growing species increased significantly in dominance at a single site, and slow-growing species increased significantly at half of our sites.

## Introduction

Tropical rain forests play a major role in the global carbon cycle: they encompass over a third of terrestrial carbon stocks [1], and they contribute approximately 30% of terrestrial net primary productivity [2]. Not only are many tropical forests under direct threat from land-use changes and logging [3–5], but it has also been suggested that pristine, apparently undisturbed rainforests may also be undergoing widespread shifts in carbon stocks and floristic composition as a result of large-scale anthropogenic environmental changes. Models suggest that plants in general, and tropical forest plants in particular, are sensitive to environmental changes such as increased atmospheric CO_2_ concentration, nitrogen deposition, temperature, drought frequency, and irradiance [6–11]. Such sensitivity could have profound implications for the future of one of earth's most critical ecosystems [12]. Field studies have reported several patterns consistent with hypothesized responses to global change [13]: increases in aboveground biomass stocks [14,15], in aboveground net primary productivity (ANPP) [16–18], in tree turnover [19], and in the dominance of fast-growing species [20,21].

These patterns of change in tropical forest and the mechanisms proposed to explain them have, however, been much debated [22–25]. An alternative explanation is that the observed changes in forest structure may be a response to natural disturbances alone. Under this second hypothesis, most forested areas in the tropics would be increasing in aboveground biomass because they were slowly recovering from past disturbances [26,27]. If this were true, this effect would be exactly offset by the large carbon losses in areas currently undergoing natural disturbances, and net ecosystem production would equal zero at the landscape scale. Neither increased ANPP nor an increase in dominance of fast-growing species would be expected under this hypothesis. Instead, the disturbance hypothesis predicts that slow-growing species would increase in dominance following a disturbance [28,29]. Here, we address these important predictions using a long-term dataset on tropical forest trees across a broad range of environmental conditions, examining both stand-level changes in biomass and changes in dominance for different guilds of tropical trees for up to 20 y of observations.

Repeated forest inventories, including detailed taxonomic identification, combined with information on species traits, enable a direct evaluation of the relationship between changes in tree species composition and aboveground carbon stores of tropical forest ecosystems. In order to evaluate the long-term changes in the dynamics and composition of tropical forests, such inventories must encompass large samples of forest, including treefall gaps [21,30]. This can be accomplished through the use of large-scale plots [31]. We used datasets from ten large (16 to 52 ha) undisturbed tropical forest dynamics plots in America (*n* = 3), Africa (*n* = 2), and Asia (*n* = 5) [32]. The dataset included over 5 million stem-diameter measurements taken between 1985 and 2005 according to a standard census protocol (see Methods, Table S1, and Text S1A). Aboveground biomass was calculated for each free-standing stem ≥ 1 cm diameter at breast height (dbh; i.e., 130 cm from the ground for most trees), and wood density. We calculated a demographic index to reflect the relative position of each taxon on a slow-growth/low-mortality to fast-growth/high-mortality axis. For each site, species were ranked using this demographic index and were partitioned into four groups with an equal number of species and roughly the same biomass. Change in biomass was defined as the annual percent change (% y^−1^) in aboveground biomass, and this was calculated in the whole plot and separately for each quartile group. For the top and the bottom quartiles, henceforth referred to as fast-growing and slow-growing groups, respectively, we assessed the statistical significance of biomass changes by bootstrapping over spatial heterogeneity.

## Results

We found that four of our plots increased significantly in aboveground biomass, three plots showed a nonsignificant trend of increasing biomass, and three showed a trend of decreasing biomass. A single plot, Sinharaja (Sri Lanka), showed a large, but not statistically significant, decline in aboveground biomass (−0.98 Mg ha^−1^ y^−1^; bootstrapped 95% confidence intervals: [−2.48, 0.40] Mg ha^−1^ y^−1^). This decline at Sinharaja was caused by the high mortality of a single shade-tolerant canopy species, Mesua nagassarium (Clusiaceae), which dominates the topographic ridges in the plot [33]: in this species, 42% of the trees ≥ 70 cm dbh (*n* = 86) and 22% of the trees ≥ 30 cm dbh (*n* = 877) died between the two censuses. Averaging over all plots, we found a significant mean aboveground biomass increase of +0.47 [0.14, 0.79] Mg ha^−1^ y^−1^ (or about +0.24 MgC ha^−1^ y^−1^). Excluding the Sinharaja plot, the other nine plots showed an average increase of (+0.63 [0.30, 0.96] Mg ha^−1^ y^−1^). These patterns were the same when we restricted our analysis to trees ≥ 10 cm dbh, as has been done in most previous studies [14,15,22,34] (Table S5 and Text S1B).

**Figure 1:**
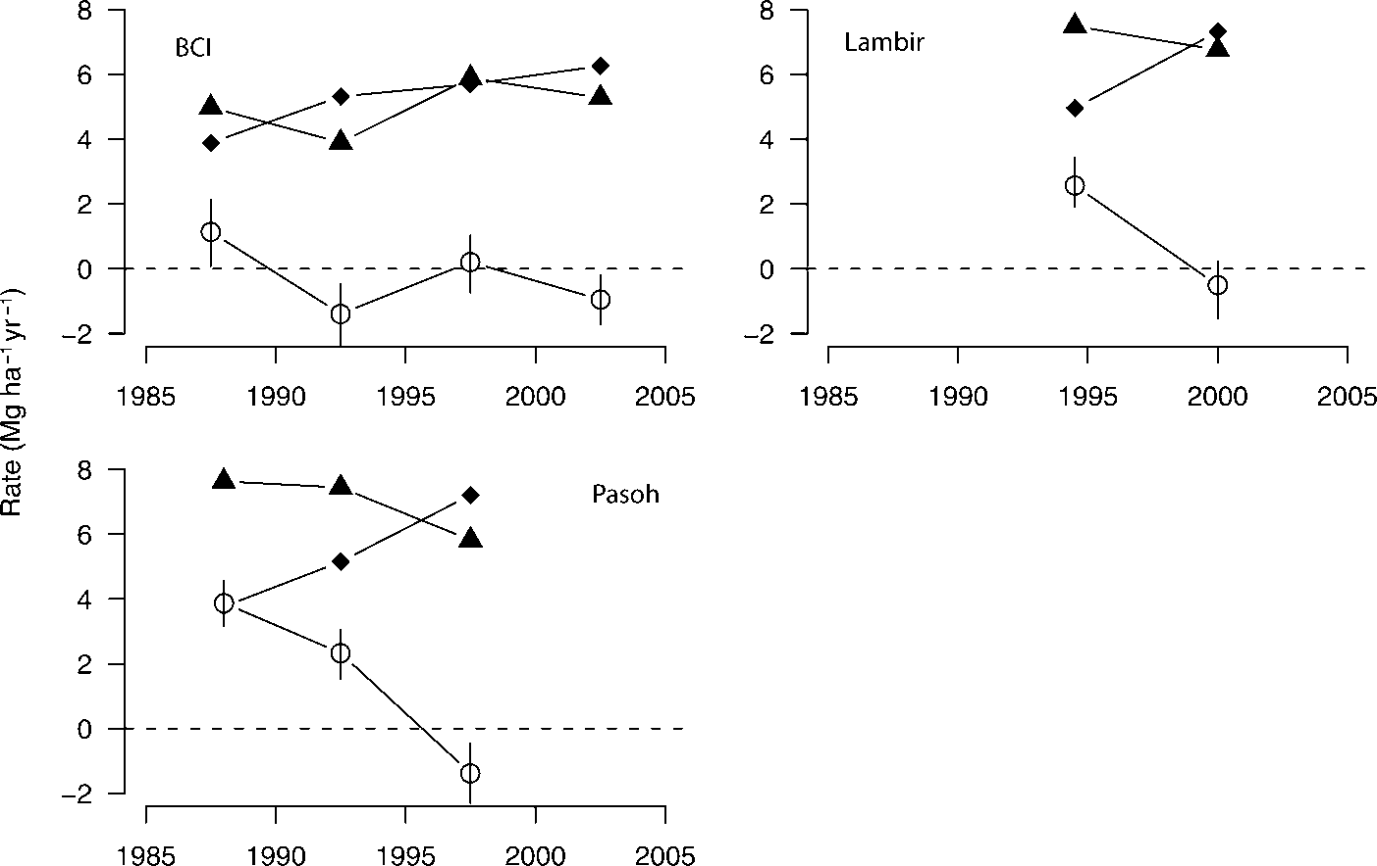
Aboveground Biomass Changes at Three Long-Term Forest Plots, BCI (Panama), Lambir, and Pasoh (Malaysia), in Mg ha^−1^ y^−1^ Triangles: aboveground biomass growth rate; diamonds: above ground biomass mortality rate; circles: net change in above ground biomass. Vertical lines represent the 95% confidence intervals computed using a spatial bootstrapping procedure. The dashed line represents the null hypothesis of no biomass change in the plots. The points are placed at the mid-points of the census intervals.

In the three plots with two or more intercensus intervals, we found that aboveground biomass did not accumulate consistently over the study period. At BCI (Panama), both significant increases and significant decreases in aboveground biomass were observed over the 20 y of study, consistent with the response of the forest to short-term disturbances, such as droughts (Figure 1). The two plots in Malaysia, Pasoh and Lambir, showed significant biomass increases between 1990 and 1995, followed by decreases between 1995 and 2000. This latter interval included a strong El Niño and regional droughts. Aboveground biomass growth rate did not consistently increase over the survey period, although it did marginally increase at BCI (Figure 1). Aboveground biomass mortality rate consistently increased at BCI, Pasoh, and Lambir across the study period.

**Figure 2:**
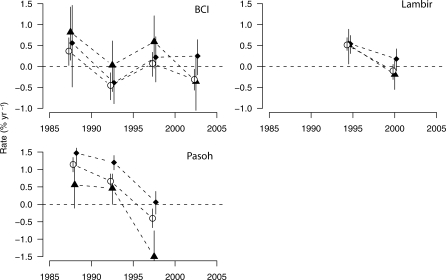
Changes in Aboveground Biomass for Fast- and Slow-Growing Species Groups, BCI, Lambir, and Pasoh, in % y^−1^ Triangles: net change in the fast-growing group; diamonds: net change in the slow-growing group; circles: stand-level net change. Demographic groups were defined based on a demographic index based on the sapling relative growth rate and the sapling mortality of the species growing in the study plots. Each point represents a separate census interval. Error bars represent the 95% confidence intervals computed using a spatial bootstrapping procedure (Methods).

Next, we explored whether biomass changes in fast-growing and slow-growing species diverged from those observed for forest stands as a whole. The fast-growing species group (species in the top quartile of the demographic index) increased significantly in biomass at only one of our plots (Table 1), Sinharaja. The slow-growing group (bottom quartile in demography) increased significantly in biomass in five of our plots. Again, Sinharaja stood out: the slow-growing group declined dramatically and significantly (−2.47 % y^−1^), reflecting the aforementioned die-off of a dominant slow-growing species. Averaging across the plots, both the fastest-growing quartile of species (0.33 [0.09, 0.55] % y^−1^), and the slowest-growing quartile (0.21 [0.02, 0.37] % y^−1^) increased significantly in biomass. Both groups increased more than the stand-level mean (+0.15 % y^−1^). As for the stand-level biomass trends, Sinharaja alone had a strong effect on the mean across plots. When this site was excluded, fast-growing species increased in biomass by only 0.17 [−0.08, 0.40] % y^−1^, not significantly different from the stand mean, whereas slow-growing species increased significantly by 0.50 [0.32, 0.65] % y^−1^. This result implies that at all sites except Sinharaja, slow-growing species increased at the expense of species growing at an intermediate rate rather than at the expense of fast-growing species. Exploring the temporal trend at the long-term plots, we found that the change in slow-growing species was consistently above the stand-level mean at Lambir and Pasoh (Figure 2). A different pattern was observed at BCI, however, where fast-growing species increased consistently more than the stand as a whole between 1985 and 2000, and then declined at the expense of slow-growing species between 2000 and 2005.

**Table 1:**
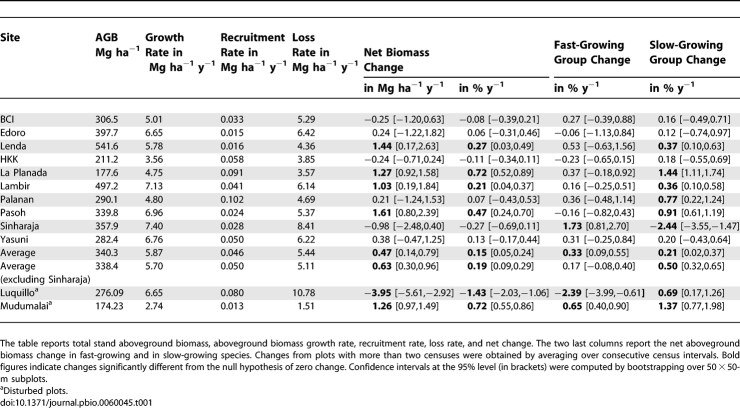
Stocks and Changes in Total Aboveground Biomass (AGB) and across Growth Groups for Ten Undisturbed Tropical Forest Plots

We next explored trends by groups based on functional traits rather than on demographic rates (Table 2). Immediately after disturbance, species increasing in abundance are generally hypothesized to have a low wood density and small seed size. As ecological succession proceeds, these species would decline at the expense of species with, on average, high wood density and large seed size [28,29]. Our results were not consistent with either scenario. Species with high wood density tended to increase in biomass at all sites except Sinharaja. Here again, this plot alone led to an overall trend for a decrease in the high wood density group (−0.12 [−0.30, 0.04] % y^−1^). At the other nine plots (excluding Sinharaja), this group increased significantly in biomass (+0.27 [0.12, 0.41] % y^−1^). In contrast, species with low wood density showed a nonsignificant change in biomass, both for all sites combined and when Sinharaja was excluded (+0.04 or −0.06 % y^−1^, respectively). Small-seeded species increased significantly in biomass (+0.37 [0.19, 0.54] % y^−1^), across all plots, and this change remained significant when Sinharaja was excluded (+0.31 [0.13, 0.48] % y^−1^). Overall, large-seeded species did not increase (+0.01 [−0.17, 0.16] % y^−1^), but they did increase significantly when Sinharaja was excluded (+0.23 [0.07, 0.37] % y^−1^). A previous study of a tropical forest near Manaus found that all the genera that increased in basal area were canopy species, whereas all declining genera were confined to the forest understory [20]. When we assigned functional groups based on maximal plant size, we found no significant changes in their dominance.

**Table 2:**
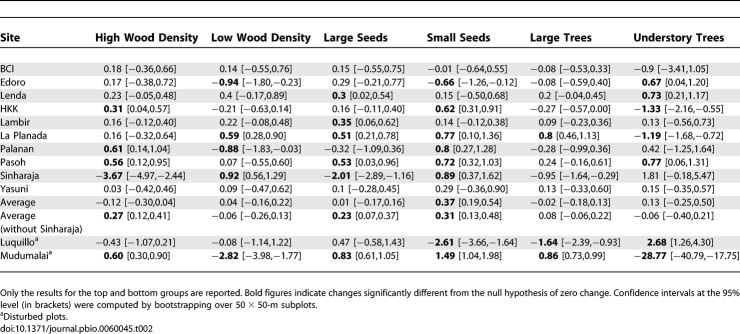
Change in Aboveground Biomass Per Species Groups Defined according to Wood Density, Seed Size, and Maximum Attainable Height (in % y^−1^)

To assess the hypothesis that disturbances lead first to decreasing biomass and increasing abundances of fast-growing species, and then to increasing biomass and increasing abundances of slow-growing species as succession occurs, we performed the same analyses with plots that experienced significant disturbances shortly before their first censuses, Luquillo (Puerto Rico) and Mudumalai (India; Text S1A). Luquillo experienced farming, selective logging, and finally, a major hurricane (Hugo) immediately before its first census [35,36]. Mudumalai underwent selective logging years prior to plot establishment [37]. The Luquillo plot decreased significantly in biomass (−1.43 [−2.03, −1.06] % y^−1^) as trees died back, damaged by Hurricane Hugo, and it also exhibited a significant increase in the abundance of fast-growing species; both patterns are consistent with predicted initial responses to disturbance. The Mudumalai plot increased significantly in biomass throughout the study period (+0.72 [0.55, 0.86] % y^−1^), and there was a consistent decline in the abundance of fast-growing species, consistent with longer-term succession following disturbance.

## Discussion

Our results from old-growth forest plots are consistent with an overall increase in aboveground biomass in tropical forests. Such an increase was previously observed in a large number of plots (*n* = 59), totalling 78 ha in size in the Amazon [15]. Here, we find the same pattern in fewer larger plots totalling 400 ha in size and spread over a broader geographical area and diversity of forest types, including a monodominant forest stand (Lenda, Democratic Republic of Congo). However, the significant mean aboveground biomass increase of +0.47 Mg ha^−1^ y^−1^ dry mass (or +0.24 MgC ha^−1^ y^−1^, assuming that 50% of dry biomass is carbon) in our plots was half as large as the +0.98 Mg ha^−1^ y^−1^ dry mass previously reported for Amazonian forests [15]. The mechanism underlying the observed increase in tropical forest biomass is still unclear. The inconsistencies of biomass growth rate over time in long-term sites do not argue strongly for a widespread increase in primary productivity in tropical forests. The increase in rate of biomass loss at these sites would instead suggest that tropical trees are growing in an increasingly unfavourable environment.

There has been some controversy in the literature about the relative merits of long-term monitoring of tropical forests based on many small plots versus a few large plots [38]. Although our study suggests that the two approaches yield similar results, these approaches should be seen as complementary, rather than competing. Our large, permanent plots are big enough to subsume the fine-scale variation created by treefall gap formation, and by site selection bias. However, they may not always appropriately sample the landscape-scale variability of the forest [22]. In contrast, existing networks of small plots cover a larger range of environmental conditions, but they currently gather sites created for other purposes than environmental monitoring. A large amount of effort has been devoted to test the possible bias related to such heterogeneous datasets [16,19,38], but complementing these tests with an independent network of large plots is important to move the debate forward. Working with large plots also is advantageous because in species-rich tropical forests, it is far easier to develop intensive botanical programs at a few sites than across networks of small scattered plots, despite recent progress in documenting spatial patterns of floristic tree diversity in the tropics [22,39].

Of our ten undisturbed plots, nine followed a dynamic consistent with the hypothesis that tropical forests are recovering from a past disturbance, with a significant increase in aboveground biomass, and a faster increase in dominance of slow-growing species relative to fast-growing species. The only exception was the Sinharaja (Sri Lanka) plot, in which an abundant canopy species, Mesua nagassarium, experienced a massive die-off during the study period. The cause of this decline is as of yet not known, although the presence of fruiting bodies of a particular fungus on the dead trees suggests a role for a pathogen. Although it has seldom been reported in large canopy trees, the massive decline of a single locally abundant species is consistent with theories of density-dependent regulation in tropical forests plants [40]. If this pattern is general across tropical forests, this would explain why many tropical forests plots are locally increasing in biomass, despite the fact that signs of large-scale past disturbances are difficult to detect.

Our results fail to support the hypothesis that fast-growing and canopy species are increasing in dominance across tropical forests [20]. We found evidence for an increase in the biomass of fast-growing species at a single site, while slow-growing species increased significantly in dominance at half of our sites. Although alternative scenarios cannot be ruled out [13], one plausible explanation is that our plots are indeed recovering from undocumented past disturbances. Successional changes in community composition are slower than changes in stand structure [41], and past meso-scale disturbances are difficult to detect in temperate and tropical forests alike [24,41,42]. For instance, careful scrutiny of the history of temperate landscapes has revealed the complexity of the interplay between natural and human disturbances [43–45], and this leads to serious uncertainties about the contribution of these environments to the global carbon cycle [46]. Even if this explanation is correct, recovery from disturbance alone is unlikely to be the only explanation for our observations of the increase in biomass and compositional shifts. It is likely that some physiological mechanism that is responding to the changing environment may also contribute. Wide-spread and long-term floristic monitoring programs in the tropics, in combination with better large-scale efforts to assess the stand structure of forests within tropical landscapes [47], are thus crucial to understanding the past, present, and future of species composition and carbon stores in tropical forests.

## Methods

### Data collection and filtering.

In each plot, all trees ≥ 1 cm in dbh were mapped, tagged, identified botanically, and had their diameters measured to the nearest millimetre in each census. There is no evidence that any of the ten main plots have been disturbed by past human activities Over 80% of the taxa, encompassing 94% of total aboveground biomass, were reliably identified to the species level. We assumed that trees that increased in diameter by more than 45 mm/y or shrank more than −5 mm/y were inaccurately measured in the field [31]. For these individuals, we corrected the diameter by assuming a mean growth rate for the individuals in the same diametric class (in millimetres, diametric size class limits were set to 10, 15, 20, 25, 30, 40, 50, 100, 200, 300, 400, 500, 600, 700, and 10,000). The same correction was applied to recruits of anomalously large diameters. The default point of measurement was at 130 cm above ground following standard forestry techniques [31]. Measurements made at different heights due to an irregularity of the bole were marked with paint. Changes in the point of measurement were recorded in the database, and they were ignored in the computation of the average dbh growth rate. However, ignoring these stems in stand-level biomass estimation would have resulted in serious underestimates, since the dbh of many of the large trees had to be measured at different heights. We then filtered the dataset as in the case of inaccurate measurements described above. Simple data corrections were performed using a computer routine, but most of these corrections were carried out manually. The dbh of all the large trees in our plots (dbh ≥ 70 cm dbh, *n* = 3,811) were manually checked. For the plots with more than two censuses, we were able to correct the anomalous dbh values more precisely, by comparing the stem dbh growth rates across census intervals. If a tree showed a dramatic change in dbh growth rate, we changed the one outside of the range (−5 mm/y, +45 mm/y) with the likely value, and updated the dbh value accordingly. This filter was applied using a computer routine, and then checked manually. In a recent work, aboveground biomass results were reported for the HKK and Pasoh sites that differ only slightly with the present figures [48]. These differences are explained by slight differences in the dataset corrections used in reference [48] and the present work. In the present work, all corrections in the raw data were performed by the lead author. All analyses were performed using the R project software, version 2.5.1 (http://www.R-project.org/).

### Estimation of tree biomass from inventories.

Aboveground biomass was calculated using a regression model that converts diameter and wood density into an estimate of total oven-dry aboveground biomass [49]. We evaluated the contribution to the aboveground carbon cycle of trees ≥ 1 cm in dbh, excluding seedlings, lianas, rattans, non-woody monocots, and rapid aboveground carbon pools (coarse woody debris, twigs, leaves, and reproductive organs). Each plot was classified into one of the following three tropical forest types: dry, moist, and wet [50,51]. The majority of the tropical forested area consists of moist forests [52]. We used the following allometric regression models for individual trees to convert the inventory data into aboveground biomass [49]:

Dry forest stands:

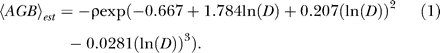



Moist forest stands:

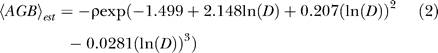



Wet forest stands:

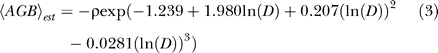



where AGB is in Mg, *D* (in cm) is the trunk diameter at breast height (130 cm above the ground, or 50 cm above any buttresses or deformities), and *r* is the corresponding wood specific gravity (oven-dry weight at 0% moisture over green volume, in g/cm^3^). In the case of multiple-stemmed trees, the allometric model was applied to each stem and summed, to provide a tree-level aboveground biomass estimate.

Palm species were often poorly estimated using the above method. We excluded climbing palms (rattans) from our analysis (abundant in all the wet Asian plots). In addition, at Pasoh and at Lambir, arborescent palms were excluded from the sampling protocol (they constitute a very small fraction of the total biomass in these forests, with the exception of *Licuala* in Lambir). In palms that showed diameter increases throughout ontogeny (genera *Socratea*, *Iriartea*, *Oenocarpus*, and *Attalea*), aboveground biomass was relatively well estimated. For the sake of consistency, we also used this model for understory palms (e.g., genera *Geonoma*, *Bactris*, and *Prestoea*), acknowledging that gain in biomass was probably largely underestimated in these genera. This is not a serious issue in most of the plots, except in the Luquillo plot, where one palm species (Prestoea acuminata) constitutes a large fraction of the estimated biomass (ca. 10% of the total). Existing allometric models for this palm [53] are based on trunk height, data that are currently unavailable for the palms in the plot. Tree fern aboveground biomass was also poorly estimated. For these reasons, we did not consider palms and tree ferns in interspecific comparisons. Note that the decrease reported in the Luquillo plot (see below) might have been exaggerated by our inaccurate estimate of the biomass dynamics in Prestoea acuminata. The missed gain might be on the order of 0.5–1.0 t/ha/y, and is insufficient to balance the observed loss.

### Statistical confidence in aboveground biomass changes.

Statistical tests within a plot were based on the computation of annual aboveground biomass change (in Mg ha^−1^ y^−1^) for each 0.25-ha subplot. Bootstrap samples of these quarter-hectare subplots were drawn 1,000 times to generate estimates of 95% confidence intervals [30]. The quadrat bootstraps were also applied to estimates of biomass change in species and species groups. Mean net changes across groups were computed by assuming the independence of the plots, and the normality of errors, described by the mean confidence interval 〈*CI*〉. If *n* samples are available, the estimated confidence interval on the mean is 〈*CI*〉 / 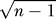 .

### Species groupings.

Demographic species groups were defined from the demographic parameters of species with at least 20 saplings (stems <5 cm and ≥1 cm dbh). Individuals of these species represented 70.5% to 95.2% of the total standing biomass (Table S3). Log-transformed sapling relative growth rate (lsRGR) and log-transformed sapling mortality rate (lsM) were positively and significantly correlated across species (*R* ^2^ = 0.208; Figure S1). Our demographic index is defined as the first principal component analysis score between lsRGR and lsM. Species were divided into quartiles based on this index; these quartiles varied in biomass because species varied in abundance (Table S3). Because some individual trees were excluded from the analyses because they were not identified to species or belonged to species that had fewer than 20 saplings, net biomass changes summed over groups did not exactly match the total stand-level net biomass change. We assumed that unclassified stems were evenly spread across the groups. Under this assumption, the net biomass change summed over groups should be equal to the net biomass change of the plot. More precisely, if a group has a biomass stock of *B_i_* (in Mg ha^−1^), a net biomass change of Δ*B_i_* (in Mg ha^−1^ y^−1^), and the entire stand has a biomass of *B* and a net biomass change of Δ*B*, the sum 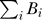 is generally smaller than *B*, because a number of species could not be classified. We then corrected Δ*B_i_* by the following formula: 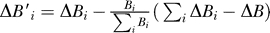 . With this correction, the sum of net biomass changes across groups 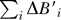 is equal to Δ*B*.

In addition to grouping species according to their demographic rates at the sapling stage, we also defined groupings using functional traits (see summary statistics in Tables S2 and S4). Wood specific gravity (referred to as wood density in the main text, for simplicity) is an important correlate of maximal growth rate and plant longevity, since species with dense wood must invest more in construction costs and are less vulnerable to stem breaks and microbial attack [54]. Wood density was estimated for the taxa based on surveys of the forestry literature [55,56]. If species-level information was unavailable, a genus-level or a family-level mean was taken. For 57 taxa, mostly in lesser-known taxonomic groups, no wood specific gravity information could be found, and a plot-level average was assumed. The contribution of these rare taxa to carbon pools and fluxes is negligible.

Seed size is an important correlate of seed production and establishment strategies [57–59]. We matched the tree taxa in the plot to the Seed Information Database (release 6.0, Oct. 2004; http://www.kew.org/data/sid). Seed mass (in grams) was based on species-level information when available (22%), otherwise on information at the genus (68%) or family (10%) level. Seed mass was log-transformed prior to statistical analyses [58].

We also used potential tree size as a final predictor of demographic success. Free-standing woody plant species vary greatly in their life history strategies, especially in comparisons among those species that complete their entire life cycles in the understory and those that do not reproduce until they emerge above the canopy. Following the precedent of recent studies [60], we estimated potential maximum tree height as 95 percentile in dbh. Understory species were defined as species whose upper 95 percentile of dbh (maxdbh95) does not reach 10 cm (for species with at least 20 individuals in a plot). Canopy species were species whose maxdbh95 ≥ 30 cm dbh.

## Supporting Information

Figure S1 Correlation between Log-Transformed Sapling Relative Growth Rate and Log-Transformed Sapling Mortality Rate across the 12 Study Plots Both relative growth rate and mortality rate are in % y^−1^. Each circle represents a species-site combination, and the solid line is the first PCA axis, which captures 20% of the variation in the two variables.
(178 KB PDF)

Table S1 Description and Environmental Characteristics of the Study Plots (52 KB DOC)

Table S2 Taxonomic Identification Level and Functional Traits in the Permanent Plots The table represents the number of taxa at three identification levels: species, genus, and family. Also shown is the number of taxa for which information on wood density and on seed weight is available at the species level, at the genus level or at the family level.
(48 KB DOC)

Table S3 Total Aboveground Biomass and Number of Individuals, Per Stand and Per Demographic Group Aboveground biomass is reported in Mg ha^−1^, number of individuals in ind. ha^−1^. Also reported is the total percentage of aboveground biomass (AGB) and of the number of individuals in the three demographic groups.
(50 KB DOC)

Table S4 Quantiles of Wood Density and of Log-Transformed Seed Mass in the 12 Study Plots Wood density is defined as oven-dry weight divided by green volume, in g cm^−3^, seed mass is in grams. Median wood density varied between 0.53 and 0.63 g cm^−3^. Median seed mass varied between 0.023 and 0.33 g.
(54 KB DOC)

Table S5 Stocks and Changes in Total Aboveground Biomass for Ten Undisturbed Tropical Forest Plots Based on Trees ≥10 cm dbh This table reports total stand aboveground biomass, aboveground biomass growth rate, recruitment rate, loss rate, and net change. Bold figures indicate changes significantly different from the null hypothesis of zero change. Confidence intervals at the 95% level (in brackets) were computed by bootstrapping over 50 × 50-m subplots.
(44 KB DOC)

Text S1 Study Plots (A) Detailed information on the 12 study plots, with an emphasis on the known disturbance history of these sites.
(B) Aboveground biomass estimation and statistical analyses based on large trees only.
(75 KB DOC)

## Abbreviations

dbh: diameter at breast height
