# Supplementary figures and images for: Assessing Evidence for a Pervasive Alteration in Tropical Tree Communities
Source: PLoS Biol. 2008 Mar 4;6(3):e45. doi: 10.1371/journal.pbio.0060045 (PMC2270308; doi:10.1371/journal.pbio.0060045)

# PCA

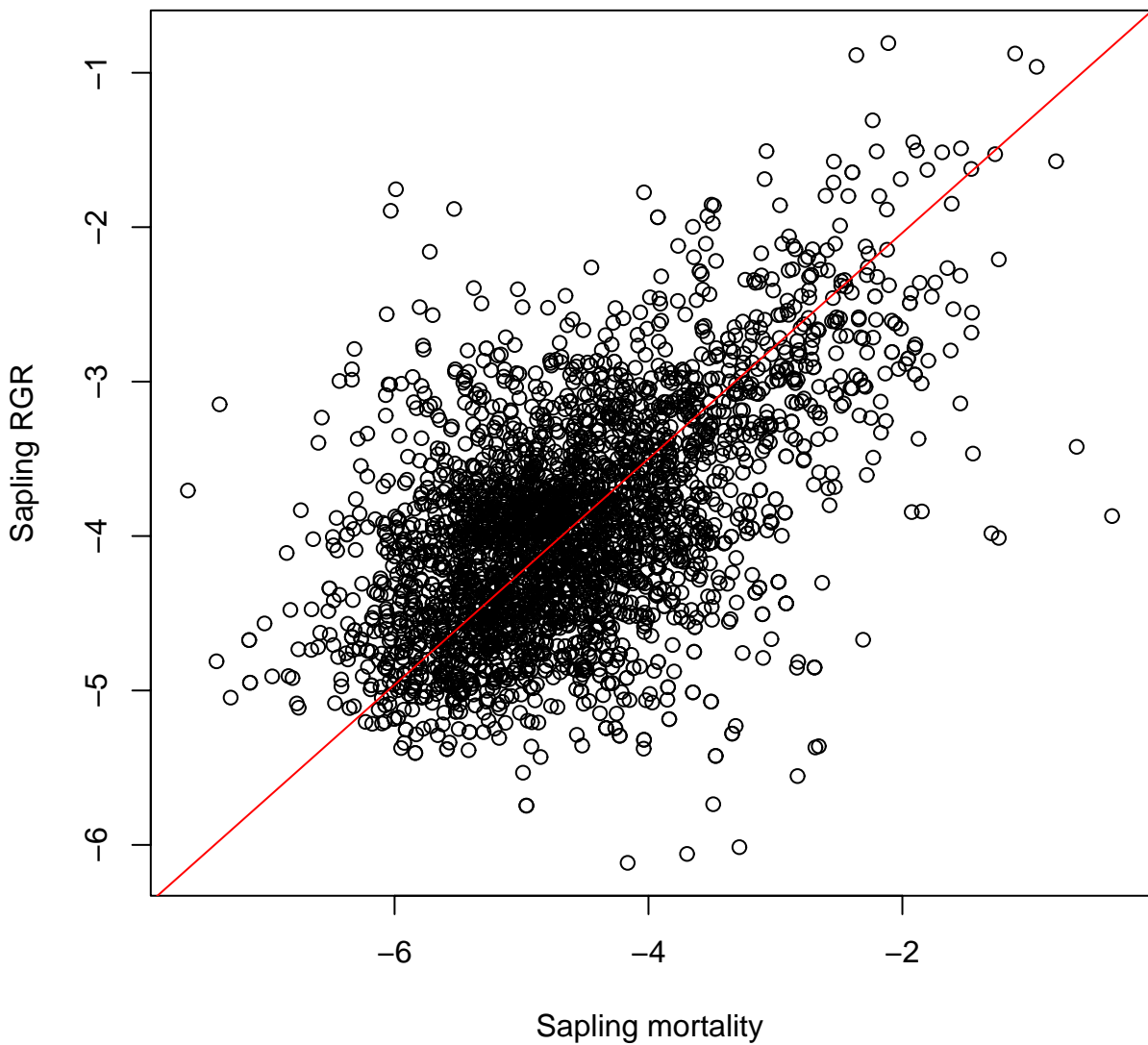

Supplement: Figure S1 — Both relative growth rate and mortality rate are in % y−1. Each circle represents a species-site combination, and the solid line is the first PCA axis, which captures 20% of the variation in the two variables. (178 KB PDF) [file pbio.0060045.sg001.pdf]
