# Supplementary material for: Assessing Evidence for a Pervasive Alteration in Tropical Tree Communities
Source: PLoS Biol. 2008 Mar 4;6(3):e45. doi: 10.1371/journal.pbio.0060045 (PMC2270308; doi:10.1371/journal.pbio.0060045)
Supplement: Table S1 — (52 KB DOC) [file pbio.0060045.st001.doc]

| Site | Country | Sampling period | Plot area (ha) | Mean no. taxa | Median rainfall (mm/yr)* | No. dry months† | Forest type |
| --- | --- | --- | --- | --- | --- | --- | --- |
|  |  |  |  |  |  |  |  |
| BCI | Panama | 1985-2005 | 50 | 303 | 2635 | 4 | moist |
| Edoro | DR Congo | 1994-1999 | 20** | 448 | 1793 | 3 | moist |
| Lenda | DR Congo | 1994-1999 | 20** | 452 | 1721 | 3 | moist |
| HKK | Thailand | 1994-1999 | 50 | 295 | 1355 | 6 | dry |
| La Planada | Colombia | 1996-2001 | 25 | 241 | ~4000 | 0 | wet |
| Lambir | Malaysia | 1992-2003 | 52 | 1322 | 2921 | 0 | moist |
| Luquillo | Puerto Rico | 1992-1995 | 16 | 142 | 2574 | 0 | wet |
| Mudumalai | India | 1988-2000 | 50 | 68 | 1553 | 6 | dry |
| Palanan | Philippines | 1999-2003 | 16 | 410 | 2607 | 3 | wet |
| Pasoh | Malaysia | 1986-2000 | 50 | 815 | 1973 | 0 | moist |
| Sinharaja | Sri Lanka | 1993-1998 | 25 | 205 | 3379 | 0 | wet |
| Yasuní | Ecuador | 1995-2000 | 24 | 1125 | 3111 | 0 | moist |

Monthly mean of daily rainfall records over the period 1950-2000 at a 1-km^2^ spatial resolution, cumulated over the months to yield an annual figure; from the Worldclim project [Error: Reference source not found]

† Number of months with less than 100 mm of rainfall.

** The Congo sites each consist of two 10-ha plots; all other sites consist of one rectangular or square plot.
