# Supplementary material for: Assessing Evidence for a Pervasive Alteration in Tropical Tree Communities
Source: PLoS Biol. 2008 Mar 4;6(3):e45. doi: 10.1371/journal.pbio.0060045 (PMC2270308; doi:10.1371/journal.pbio.0060045)
Supplement: Table S2 — The table represents the number of taxa at three identification levels: species, genus, and family. Also shown is the number of taxa for which information on wood density and on seed weight is available at the species level, at the genus level or at the family level. (48 KB DOC) [file pbio.0060045.st002.doc]

|  | Identification level | | | Wood density | | | Seed mass | | |
| --- | --- | --- | --- | --- | --- | --- | --- | --- | --- |
| site | species | genus | family | species | genus | family | species | genus | family |
| BCI | 311 | 6 | 0 | 152 | 134 | 31 | 160 | 135 | 21 |
| Edoro & Ituri | 429 | 10 | 4 | 79 | 193 | 183 | 35 | 276 | 130 |
| HKK | 286 | 17 | 0 | 130 | 150 | 21 | 61 | 196 | 43 |
| Lambir | 1015 | 175 | 0 | 446 | 696 | 48 | 91 | 859 | 231 |
| La Planada | 92 | 124 | 17 | 16 | 158 | 47 | 7 | 154 | 62 |
| Luquillo | 147 | 1 | 0 | 75 | 57 | 15 | 54 | 80 | 12 |
| Mudumalai | 70 | 1 | 0 | 42 | 23 | 9 | 33 | 36 | 2 |
| Palanan | 264 | 75 | 2 | 97 | 210 | 32 | 25 | 249 | 64 |
| Pasoh | 772 | 50 | 0 | 438 | 332 | 49 | 97 | 544 | 176 |
| Sinharaja | 199 | 7 | 0 | 29 | 156 | 21 | 20 | 154 | 31 |
| Yasuní | 774 | 328 | 51 | 303 | 700 | 139 | 133 | 809 | 205 |
|  |  |  |  |  |  |  |  |  |  |
| Total | 4359 | 794 | 74 | 1807 | 2809 | 595 | 716 | 3492 | 977 |
