# Supplementary material for: Assessing Evidence for a Pervasive Alteration in Tropical Tree Communities
Source: PLoS Biol. 2008 Mar 4;6(3):e45. doi: 10.1371/journal.pbio.0060045 (PMC2270308; doi:10.1371/journal.pbio.0060045)
Supplement: Table S3 — Aboveground biomass is reported in Mg ha−1, number of individuals in ind. ha−1. Also reported is the total percentage of aboveground biomass (AGB) and of the number of individuals in the three demographic groups. (50 KB DOC) [file pbio.0060045.st003.doc]

| Site | Total AGB | AGB slow-growing | AGB intermediate-growing | AGB fast-growing | % AGB in groups |  | Nb of individuals | Nb slow-growing | Nb intermediate -growing | Nb fast-growing | % indiv. in groups |
| --- | --- | --- | --- | --- | --- | --- | --- | --- | --- | --- | --- |
|  |  |  |  |  |  |  |  |  |  |  |  |
| BCI | 306.89 | 74.19 | 125.71 | 47.57 | 80.64 |  | 223974 | 77585 | 130121 | 12483 | 98.31 |
| Edoro | 400.37 | 44.33 | 196.11 | 88.79 | 82.23 |  | 154456 | 20643 | 104813 | 26075 | 98.11 |
| Lenda | 546.52 | 418.40 | 50.59 | 48.21 | 94.64 |  | 133631 | 27367 | 91294 | 12433 | 98.10 |
| HKK | 210.36 | 24.08 | 97.56 | 20.47 | 67.55 |  | 72604 | 15576 | 43128 | 7433 | 91.09 |
| Lambir | 498.20 | 67.75 | 235.74 | 166.52 | 94.34 |  | 373012 | 121221 | 172317 | 66517 | 96.53 |
| LaPlanada | 181.33 | 51.07 | 93.82 | 27.68 | 95.17 |  | 102426 | 24279 | 52736 | 19813 | 94.53 |
| Palanan | 288.29 | 40.71 | 111.59 | 101.11 | 87.90 |  | 81038 | 23874 | 33161 | 10469 | 83.30 |
| Pasoh | 344.39 | 72.56 | 154.90 | 90.18 | 92.23 |  | 324764 | 95184 | 161044 | 43717 | 92.36 |
| Sinharaja | 353.07 | 112.30 | 175.51 | 47.27 | 94.90 |  | 193353 | 71199 | 96895 | 23084 | 98.88 |
| Yasuni | 282.87 | 43.44 | 109.53 | 46.36 | 70.47 |  | 138458 | 38910 | 63172 | 21468 | 89.23 |
