# Supplementary material for: Assessing Evidence for a Pervasive Alteration in Tropical Tree Communities
Source: PLoS Biol. 2008 Mar 4;6(3):e45. doi: 10.1371/journal.pbio.0060045 (PMC2270308; doi:10.1371/journal.pbio.0060045)
Supplement: Table S4 — Wood density is defined as oven-dry weight divided by green volume, in g cm−3, seed mass is in grams. Median wood density varied between 0.53 and 0.63 g cm−3. Median seed mass varied between 0.023 and 0.33 g. (54 KB DOC) [file pbio.0060045.st004.doc]

| Site | Wood density (g cm^-3^) | | | | |  | Seed mass (g, log-transformed) | | | | |
| --- | --- | --- | --- | --- | --- | --- | --- | --- | --- | --- | --- |
|  | 0% | 25% | 50% | 75% | 100% |  | 0% | 25% | 50% | 75% | 100% |
|  |  |  |  |  |  |  |  |  |  |  |  |
| BCI | 0.13 | 0.45 | 0.56 | 0.65 | 0.89 |  | -11.74 | -5.03 | -2.76 | -0.93 | 3.82 |
| Edoro & Ituri | 0.22 | 0.54 | 0.63 | 0.68 | 0.94 |  | -8.65 | -3.87 | -1.96 | -0.43 | 3.67 |
| HKK | 0.21 | 0.48 | 0.58 | 0.65 | 0.99 |  | -8.95 | -4.18 | -2.48 | -1.02 | 3.67 |
| Lambir | 0.21 | 0.51 | 0.59 | 0.66 | 0.93 |  | -10.72 | -2.71 | -1.10 | 0.22 | 3.67 |
| La Planada | 0.16 | 0.47 | 0.53 | 0.62 | 0.84 |  | -11.51 | -7.02 | -3.74 | -1.04 | 2.93 |
| Luquillo | 0.13 | 0.49 | 0.60 | 0.68 | 0.90 |  | -10.13 | -5.40 | -2.92 | -1.21 | 2.80 |
| Mudumalai | 0.21 | 0.50 | 0.60 | 0.68 | 0.87 |  | -9.21 | -4.34 | -2.64 | -0.92 | 2.80 |
| Palanan | 0.21 | 0.48 | 0.57 | 0.65 | 0.84 |  | -11.68 | -3.83 | -1.96 | -0.19 | 3.22 |
| Pasoh | 0.23 | 0.52 | 0.59 | 0.68 | 0.92 |  | -11.51 | -2.75 | -1.38 | 0.06 | 3.67 |
| Sinharaja | 0.29 | 0.52 | 0.59 | 0.68 | 0.93 |  | -10.98 | -3.11 | -2.12 | -0.14 | 2.90 |
| Yasuni | 0.13 | 0.51 | 0.60 | 0.68 | 1.08 |  | -11.87 | -3.87 | -1.76 | -0.51 | 3.57 |
|  |  |  |  |  |  |  |  |  |  |  |  |
