# Supplementary material for: Assessing Evidence for a Pervasive Alteration in Tropical Tree Communities
Source: PLoS Biol. 2008 Mar 4;6(3):e45. doi: 10.1371/journal.pbio.0060045 (PMC2270308; doi:10.1371/journal.pbio.0060045)
Supplement: Table S5 — This table reports total stand aboveground biomass, aboveground biomass growth rate, recruitment rate, loss rate, and net change. Bold figures indicate changes significantly different from the null hypothesis of zero change. Confidence intervals at the 95% level (in brackets) were computed by bootstrapping over 50 × 50-m subplots. (44 KB DOC) [file pbio.0060045.st005.doc]

|  | AGB | Gain | Recruitem. | Loss | Net change |
| --- | --- | --- | --- | --- | --- |
|  |  |  |  |  |  |
| BCI | 292.17 | 4.19 | 0.53 | 4.97 | -0.25 [-1.15,0.62] |
| Edoro | 375.06 | 5.80 | 0.60 | 6.14 | 0.26 [-1.34,1.73] |
| Lenda | 525.34 | 5.39 | 0.21 | 4.18 | **1.43** [1.08,1.74] |
| HKK | 202.70 | 2.96 | 0.50 | 3.59 | -0.14 [-0.61,0.33] |
| LaPlanada | 160.04 | 3.53 | 0.82 | 2.94 | **1.41** [0.10,1.82] |
| Lambir | 479.13 | 6.37 | 0.47 | 5.87 | **0.97** [0.13,2.61] |
| Palanan | 276.27 | 3.83 | 0.73 | 4.57 | -0.01 [-1.44,1.35] |
| Pasoh | 319.98 | 6.03 | 0.63 | 5.11 | **1.55** [0.68,2.31] |
| Sinharaja | 336.87 | 6.36 | 0.74 | 8.14 | -1.05 [-2.54,0.44] |
| Yasuni | 262.93 | 5.61 | 0.80 | 5.93 | 0.47 [-0.42,1.27] |
|  |  |  |  |  |  |
| Average | 323.05 | 5.01 | 0.60 | 5.14 | 0.46 [0.13,0.78] |
|  |  |  |  |  |  |
