# Supplementary material for: Assessing Evidence for a Pervasive Alteration in Tropical Tree Communities
Source: PLoS Biol. 2008 Mar 4;6(3):e45. doi: 10.1371/journal.pbio.0060045 (PMC2270308; doi:10.1371/journal.pbio.0060045)
Supplement: Text S1 — (A) Detailed information on the 12 study plots, with an emphasis on the known disturbance history of these sites. (B) Aboveground biomass estimation and statistical analyses based on large trees only. (75 KB DOC) [file pbio.0060045.sd001.doc]

**Supplementary Information**

Assessing evidence for a pervasive alteration in tropical tree communities.

Jérôme Chave et al.

**A. Study plots**

Detailed information on the study plots is provided in Table S1. None of the 10 plots used in the main analysis has undergone recent and massive disturbance, either due to humans or to natural phenomena [1].

- The BCI plot (Panama), part of the Barro Colorado Nature Monument (created in 1923), has undergone a high sapling mortality due to the intense El Niño of 1983. The vast majority of this plot is old growth forest and it has remained continuously forested for well over 500 yrs, except ca. 2 ha of forest (4% of the total area) that was cleared during the 19^th^ century, and that is now dominated by the tree species *Gustavia superba*. Because this plot was not dominated by secondary growth forest species, we kept it in the analyses.
- The Edoro and Lenda plots (RD Congo) are part of the Okapi Faunal Reserve, created in 1992, have no record of logging, or natural disturbance within the plots.
- The HKK plot (Thailand), in the Huai Kha Khaeng Wildlife Sanctuary (created in 1972), has never been logged, but surface fires have been reported during the dry season in 1991 and 1998, and elephants are present in and around the plot.
- The La Planada plot (Colombia), in the La Planada Nature Reserve (created in 1982), has undergone local windthrows over small areas, and limited palmito harvest (*Prestoea acuminata*) has occurred over 25 years ago (*unpublished information from*: M. Achicando, T. Díaz Paz and H. Caguasango, residents of the San Isidro path and old workers of the Buenos Aires farm, and G. Cantillo employee of the La Planada Natural Reserve from its creation in 1982).
- The Lambir plot, in the Lambir Hills National Park (established in 1975) has had landslips (notably in 1963), and El Niño related droughts. Although it is relatively close to the former Miri-Kunching truck road (now abandoned), there is no record of human logging activities within the plot.
- The Palanan plot (Philippines), in the Palanan Reserve Area, is in the typhoon belt. It has undergone the effects of a typhoon (Imbubo) in July 2003, that defoliated trees on the windward side of the plot (ca. 5 ha). However, Co et al. (2004) [2] report that ‘powerful typhoons (class three and above) strike every few years. The exact frequency of these typhoons is not clear but the living memory of Palanan residents suggests that typhoons destructive to the forest occur every three years or so’. Because of the recurrent nature of this disturbance, the forest is likely in equilibrium with these disturbances. Co et al. (2004) also mention that ‘deep logging trails can be found in the [Palanan] plot itself, although the amount of trees extracted from the plot appears to be low’ [2].
- The Pasoh plot (peninsular Malaysia) is located in an area gazetted in 1917 as the largest forest reserve in the Malayan system, and the core part in which the plot lies has remained undisturbed forest since then. The plot has been initiated in 1961 [3] extended in 1970, and established as a 50-ha permanent plot in 1986 [4]. One of the notable local disturbances in the understorey of the Pasoh forest is the action of wild pigs [5].
- The Sinharaja plot (Sri Lanka) belongs in a forest tract protected since 1907 as a result of a Forest Ordinance. In 1978, the forest was declared an International Man and the Biosphere Reserve. There is no evidence of human disturbance within the plot. However, botanical evidence suggests occasional catastrophic disturbances in this forest, presumably by wind [6].
- The Yasuni plot (Ecuador) is part of Yasuni National Park and Biosphere Reserve. It was initiatied in 1995. Archeological remains suggest a human presence at this site 500-1000 yrs ago [7]. A 1-ha area (4% of the total area) near the southwest corner of the plot was a heliport opened well before 1990 [7], and that is now dominated by pioneer tree species (*Cecropia ficifolia*, *Cecropia sciadophylla*). We therefore excluded this disturbed area from our analysis, reducing the plot size to 24 ha.

Two more plots used in the present study and included in the CTFS network, did undergo massive disturbances in a recent past.

- The Luquillo 16-ha plot is located in the Luquillo Experimental Forest (established in 1956). Of the 16 ha in the plot, only 5.25 ha has always remained as a closed-canopy forest, although it was selectively logged in the 1940s, and until 1953. The rest was either clear-cut or intensively logged prior to 1932 [8]. In addition, this plot underwent a massive disturbance just before the first census due to Hurricane Hugo (September 1989), resulting in a mortality of 9% of the trees, defoliation of over 50% of the trees. The Large numbers of fast growing trees recruited after the hurricane were recorded in the first census, and the high mortality of these trees was recorded in the second census used in this study. The large abundance of palms at this site is probably a result of the action of hurricanes [9].
- The Mudumalai plot, in the Mudumalai Wildlife Sanctuary (originally established in 1940) is regenerating after several cycles of logging for the major timber species (including teak). Logging activities opened the forest to grasses and most likely increased the frequency of intense ground fires, which occurred in the plot in 1989, 1991, 1992, 1994, and 1996. The plot is also frequently disturbed by mammals, especially elephants.

**B. Analyses based on large trees only**

Most previous studies of tropical forest aboveground biomass changes based on tree diameter census data have restricted their analysis to trees greater than 10 cm in dbh [10], in effect assuming that recruiting trees had a dbh of 0 in the previous census. However, it is most likely that trees recruiting to 10 cm were already present in the plot five years before, and they probably already had a diameter close to 10 cm [11,12]. Thus the contribution of recruits to overall biomass change in these earlier may have resulted in an overestimation of aboveground biomass changes in tropical forests [13]. To allow for comparison with these previous studies, and to evaluate their potential bias, we repeated our analyses using only stems ≥ 10 cm dbh. The assumption that recruiting trees had a diameter of zero cm in the previous census yielded results comparable to the estimation based on all trees ≥ 1 cm dbh, confirming the assumption made elsewhere [11]. Aboveground biomass stocks were within 90% to 97% of the estimates reported in the main text (minimum at La Planada, Colombia). Both total aboveground biomass gain (summing tree growth plus recruitment), and aboveground biomass loss were consistently around 95% of the total, except again at La Planada (89%, and 82%, respectively). The mean net change remained significant (+0.46 [0.13,0.78] Mg ha^-1^ yr^-1^).

We interpret this result is as follows. Assuming that trees that just recruited to 10 cm dbh had a zero dbh in the previous census substantially overestimates the true biomass gain in these individual stems. By assuming that recruiting trees have grown from 0 cm to 10 cm during the census interval, an average biomass gain due to recruitment compensates almost exactly the missing gain.

**Supplementary references**

1. Losos EC, Leigh, E.G. Eds (2004) *Tropical Forest Diversity and Dynamism* (Chicago University Press, Chicago).
2. Co LL, Lagunzad DA, LaFranckie JV, Bartolome NA, Molina JE et al. Palanan Forest Dynamics Plot, Philippines. In Losos EC, Leigh Jr., EG (eds). *Tropical Forest Diversity and Dynamism*.(Chicago University Press, 2004).
3. Wong YK, Whitmore TC (1970) On the influence of soil properties on species distribution in a Malayan lowland dipterocarp rain forest. Malay Forest 33:42-54.
4. Ashton PS, Okuda T, Manokaran N (2003) Pasoh forest, past and present. Pp 1-14 in T. Okuda et al. (eds.) *Pasoh: Ecology of a Lowland Rain Forest in South-East Asia*. (Springer-Verlag, Tokyo).
5. Ickes K, DeWalt SJ, Thomas SC (2003) Resprouting of woody saplings following stem snap by wild pigs in a Malaysian rain forest. J Ecol 91:222-233.
6. Ashton PS, Gunatilleke CVS (1987) New light on the plant geography of Ceylon I. Historical plant geography. J Biogeogr 14:249-285.
7. Valencia R, Condit R, Foster RB, Romoleroux K, Villa Munoz G et al. (2004) Yasuni Forest Dynamics Plot, Ecuador. In Losos EC, Leigh Jr. EG (eds). *Tropical Forest Diversity and Dynamism*.(Chicago University Press).
8. Thompson J, Brokaw N, Zimmerman JK, Waide RB, Everham III, EM et al. (2002) Land-use history, environment and tree composition in a tropical forest. Ecol Appl 12:1344-1363.
9. Zimmerman JKH, Covich AP (2007) Damage and recovery of riparian sierra palms after Hurricane Georges: influence of topography and biotic characteristics. Biotropica 39:43-49.
10. King DA, Davies SJ, Noor NSM (2006) Growth and mortality are related to adult tree size in a Malaysian mixed dipterocarp forest. For Ecol Manage 223:152-158.
11. Phillips OL, Malhi Y, Vinceti B, Baker T, Lewis SL et al. (2002) Changes in the biomass of tropical forests: evaluating potential biases Ecol Appl 12:576-587.
12. Clark DA (2002) Are tropical forests an important carbon sink? Reanalysis of the long-term plot data Ecol Appl 12:3-7.
13. Clark DA (2004) Sources or sinks? The response of tropical forests to current and future climate and atmospheric composition. Phil Trans Roy Soc B 359:477-491.
14. Hijmans RJ, Cameron SE, Parra JL, Jones PG, Jarvis A (2005) Very high resolution interpolated climate surfaces for global land areas. Int J Clim 25:1965-1978.
